# The effects of Fastin®-RR, a commercial weight loss product, on body weight and body composition, resting hemodynamics and psychological mood in overweight and obese persons participating in an eight week weight loss program

**DOI:** 10.1186/1550-2783-11-S1-P20

**Published:** 2014-12-01

**Authors:** Patrick L Jacobs

**Affiliations:** 1 Superior Performance Research, Miami, Florida, USA

## Background

Over 69% of all Americans are classified as either overweight or obese. This epidemic has fueled the growth of over the counter weight loss products into one of the largest categories of nutritional supplements. However, few of these products have undergone controlled clinical trials to substantiate the respective advertising claims. Furthermore, few weight loss products have been properly examined with a placebo/controlled design in persons that are overweight or obese. The purpose of the present investigation was to examine the effects of the commercial weight loss/energy product, Fastin®-RR (High-Tech Pharmaceuticals, Inc., Norcross, GA) in overweight and obese men and women participating in a weight loss program.

## Methods

This study was carried out with seventy two men (n=36) and women (n=36) that voluntarily participated. Inclusion criteria included classification as either overweight or obese based on testing with BodPod. All research participants participated in the same eight week weight loss program including general recommendations for exercise and dietary modifications. Subjects were randomly assigned to receive one of three supplement conditions for the study period including FastinR-RR (FAS), 300 mg caffeine anhydrous (CAF), and cellulose placebo condition (PL) and were instructed to take one serving in the morning and one serving at midday. Study outcomes were determined at baseline and after 4wks and 8wks of study. Body weight and composition were determined using BodPod. Resting hemodynamic activity (HR, BP) was examined using an automated system (Dynamap 1846SX; Critikon Company LLC, Tampa, Fla). The profile of mood states (POMS-SF) was used to determine psychological measures. Only the 59 participants that completed all testing sessions were included in the statistical analyses (FAS, n=20; CAF, n=19; PL=20). Primary analyses of outcome variables were performed using changes scores from baseline to 8wks. Statistical analyses were conducted using one-way ANOVA with the accepted level of significance set at p<0.05. Consent to publish the results was obtained from all participants.

## Results

Analyses indicated that total weight loss over the 8wk period was significantly greater with FAS (-8.0±6.5 lbs) compared with PL (-2.6±4.8 lbs) and compared with CAF (-3.0±5.5 lbs) (p’s < 0.05).

The FAS group also displayed statistically greater reduction in body fat (-6.3±7.3 lbs) compared with PL (-1.3±6.7 lbs) p<0.05). There were no statistically significant changes in resting HR or BPs in any of the three study groups (FAS, CAF, PL) over the 8wk study period. Analyses showed a significant main effect of time (baseline to 8wks) in values of Total Mood Disorder indicating an overall reduction across the three study groups. There were no other significant changes detected in POMS measures over the 8wk study period in any of the three study groups.

## Conclusion

These findings indicate that total body weight and body fat weight are significantly reduced with Fastin®-RR (p’s<0.05). The changes in body weight and body fat with FAS were significantly greater than those observed with either CAF or PL. These findings showed these beneficial changes in body composition occurred without negative effects on resting hemodynamics or psychological mood.

